# A survey weighted analysis of HPTN 071 (PopART) primary outcome of HIV incidence

**DOI:** 10.1186/s12981-025-00720-0

**Published:** 2025-03-07

**Authors:** Timothy M. Skalland, Jean de Dieu Tapsoba, Sahar Z. Zangeneh, Sian Floyd, Helen Ayles, Peter Bock, Sarah Fidler, Susan H. Eshleman, Richard J. Hayes, Deborah Donnell, the HPTN 071 (PopART) Study Team

**Affiliations:** 1 Vaccine and Infectious Disease Division, Fred Hutchinson Cancer Center, 1100 Fairview Ave N, Mail Stop M2-C200, 206-667-118698109, Seattle, WA USA; 2 Center for Official Statistics, RTI International, Research Triangle Park, USA; 3 University of Washington, Seattle, USA; 4 London School of Hygiene and Tropical Medicine, London, UK; 5 School of Medicine, University of Zambia, Lusaka, Zambia; 6 Department of Paediatrics and Child Health, The Desmond Tutu Tuberculosis Centre, Stellenbosch University, Cape Town, South Africa; 7 Department of Medicine, Imperial College, London, UK; 8 Johns Hopkins University School of Medicine, Baltimore, USA

**Keywords:** PopART, HIV, Incidence, Weighting, Survey, UTT, Sampling

## Abstract

**Introduction:**

HPTN 071 (PopART) implemented a comprehensive HIV prevention package which aimed to reduce HIV incidence within 21 communities of Zambia and South Africa: Arm A, PopART intervention of universal HIV testing and treatment; Arm B, PopART intervention of universal HIV testing with ART provided according to local guidelines; and Arm C, standard of care. Analyses so far have not accounted for the sampling design of the enrolled cohort. We performed a sample-weighted re-analysis of the primary outcome of the PopART trial to derive a population-based estimate of the intervention effect.

**Methods:**

Enrollment used a two-stage sampling design: household and adult participant within each household. We constructed post-stratification weights to match the age and sex distribution of the target population in these communities. Weighted Poisson regression was used to estimate community-level HIV incidence. The PopART intervention effect was estimated using log-transformed community-level incidence estimates in an ANCOVA model.

**Results:**

The analysis based on community-level incidence shows a 25% reduction in incidence for Arm B communities compared to standard of care (RR: 0.75, 95% CI: 0.56–1.02, p = 0.06) while Arm A communities show no difference in HIV incidence compared to standard of care (RR: 1.08, 95% CI: 0.81–1.46, p = 0.56).

**Conclusions:**

Our re-analysis shows 25% reduction in HIV incidence comparing Arm B to Arm C communities. No effect was observed comparing Arm A communities to Arm C communities. These results align with the primary results of the PopART trial.

ClinicalTrials.gov number, NCT01900977, HPTN 071 [PopArt].

**Supplementary Information:**

The online version contains supplementary material available at 10.1186/s12981-025-00720-0.

## Introduction

From 2013 to 2018, HPTN 071 (PopART) implemented a comprehensive HIV prevention package which aimed to increase ART coverage through household-based HIV testing and facilitated linkage to HIV care throughout the community and thus reduce HIV incidence within 21 communities of Zambia and South Africa [1]. The main objective was to compare HIV incidence among the three study arms: Arm A, PopART intervention of universal HIV testing with universal ART; Arm B, PopART intervention of universal HIV testing with ART provided according to local guidelines (universal ART beginning in 2016); and Arm C, standard of care. The 21 communities were grouped into 7 matched triplets and the three communities in each triplet were randomly assigned to study arms. To measure the impact of the PopART intervention, the trial enrolled research participants in a population cohort (PC) consisting of one randomly selected adult 18–44 years of age recruited from a random sample of approximately 2000 households in each community. Each enrolled participant had a baseline visit (labelled PC0) and three annual follow-up visits (labelled PC12, PC24 and PC36). Annual study visits included, but were not limited to, a collection of plasma for laboratory-based HIV testing. The PopART intervention (combined across two intervention arms) showed a 20% reduction [2] in HIV incidence compared to standard of care.

The analyses of the impact of the PopART intervention have so far not accounted for the sampling design of the PC, specifically how participation in the PC differed by sex and age. While the primary analysis of PopART did account for differences between communities in the age-sex distribution within the enrolled cohort, it did not perform any adjustment for the age-sex distribution in the general population compared to the distribution within the cohort. The PC oversampled women, particularly young women, across nearly all 21 communities. Thus, the estimates of the impact of the PopART intervention were more influenced by the impact of the intervention in women in these communities than men. Moreover, the trial found the reduction in HIV incidence was larger for men than women in these communities, particularly men aged 25–44 years.

Here, we perform a sample-weighted re-analysis of the primary outcome of the HPTN 071 (PopART) trial, using the PC sampling design and data collected as part of PopART Intervention delivery, to derive a population-based estimate of the intervention effect on HIV incidence.

## Methods

This analysis includes participants enrolled in the first year of the trial (PC0 cohort) since design weights were only known for this cohort. The PC cohort used a two-stage sampling design: household (HH) sampling and sampling of an adult participant (18–44 years old) within each household. Household sampling began via a “household census” conducted in 2013. Households were randomly permuted into three groups per community: active list of 3125 households, reserve list of 1875 households, and the rest not sampled. All households in the active list were approached; if the target sample size was not reached using the active list, then households in the reserve list were approached until 2000–2500 participants were recruited, or the study round ended. Let N_h_ denote the number of households in community *h*. Let n_h_ = n_hActive_ + n_hReserve_ be the total number of households selected to be sampled in community *h* and let r_h_ = r_hActive_ + r_hReserve_ be the total number of households ‘responding’ in community *h*, i.e., approached, enumerated, and with an eligible member. Because of the random permutation of the list of households, we assume nonresponding households are missing completely at random. Even though the households in the active list were all approached, we will have the same structure for the probability of a sampled and responding household in active and reserve lists.

1 $${\text{P}}\left({\text{H}}{\text{H}}_{\text{ih}}\text{ sampled and H}{\text{H}}_{\text{ih}}\text{ responds and H}{\text{H}}_{\text{ih}}\text{ in active list}\right)\text{ = }\frac{{\text{n}}_{\text{hActive}}}{{\text{N}}_{\text{h}}}{*}\frac{{\text{r}}_{\text{hActive}}}{{\text{n}}_{{\text{hActiv}}{\text{e}}}}{=} \frac{{\text{r}}_{\text{hActive}}}{{\text{N}}_{\text{h}}}$$

2 $${\text{P}}\left({\text{H}}{\text{H}}_{\text{ih}}\text{ sampled and H}{\text{H}}_{\text{ih}}\text{ responds and H}{\text{H}}_{\text{ih}}\text{ in reserve list}\right) = \frac{{\text{n}}_{\text{hReserve}}}{{\text{N}}_{\text{h}}}{*}\frac{{\text{r}}_{\text{hReserve}}}{{\text{n}}_{\text{hReserve}}}= \frac{{\text{r}}_{\text{hReserve}}}{{\text{N}}_{\text{h}}}$$

The base sampling weight (${\text{b}}_{\text{ih}})$ for household* i* in community *h* is the inverse of the expressions in Eqs. (1) and (2).

3 $${\text{b}}_{\text{ih}}\text{=} \left\{\begin{array}{c}\frac{{\text{N}}_{\text{h}}}{{\text{r}}_{\text{hActive}}} \, {\text{i}}{\text{f}} \, {\text{H}}{\text{H}}_{\text{ih}} \, {\text{i}}{\text{n}} \, {\text{a}}{\text{c}}{\text{t}}{\text{i}}{\text{v}}{\text{e}} \, {\text{l}}{\text{i}}{\text{s}}{\text{t}}\\ \frac{{\text{N}}_{\text{h}}}{{\text{r}}_{\text{hReserve}}} \, {\text{i}}{\text{f}} \, {\text{H}}{\text{H}}_{\text{ih}} \, {\text{i}}{\text{n}} \, {\text{r}}{\text{e}}{\text{s}}{\text{e}}{\text{r}}{\text{v}}{\text{e}} \, {\text{l}}{\text{i}}{\text{s}}{\text{t}}\end{array}\right.$$

The study team randomly selected one eligible participant per responding household. The individual level weight for individual *j* in household *i* of community *h* (${w}_{hij}$) was the inverse of the probability of an individual and their household being selected (${\pi }_{hij}$).

$${\Pi}_{\text{hij}}= \text{ P}\left(\text{individual }{\text{j}}\text{ in HH }{\text{i}}\text{ of community }{\text{h}}\text{ being selected}\right)$$

$$= \text{ P} \left(\text{individual }{\text{j}}\text{ selected }\right|\text{ HH }{\text{i}}\text{ of community }{\text{h}}\text{ selected) P}\left(\text{HH }{\text{i}}\text{ in community }{\text{h}}\text{ selected}\right)$$

$${=}\left(\frac{1}{\text{number of eligible people in HH }{\text{i}}} \, \right)\text{*} \frac{1}{{\text{b}}_{\text{ih}}}$$

So,

$${{\text{w}}_{\text{hij}}}= 1/{\pi}_{\text{hij}}= \text{ } {\text{b}}_{\text{ih}}\times \text{ (number of eligible people in HH }{\text{i}}\text{ of community }{\text{h}}\text{)}$$

After the PC0 sampling concluded, study leadership noticed an unusually large occurrence of single person enumerated households, which effectively eliminated the random selection of an eligible individual within those households. It was determined that household enumeration was not thoroughly documented at PC0 and thus a re-enumeration was performed during the second year (PC12). For this analysis, we used updated PC12 household enumeration data for single person households, where available. This resulted in 40% of the PC0 single-person households getting an updated enumeration in PC12.

We used post-stratification on sex and age group to adjust for survey nonresponse. Age and sex were chosen since they were the only characteristics collected on both enrolled and non-enrolled members at household enumeration by the study team and using these would mirror the primary analysis of PopART which adjusted for age and sex. We constructed post-stratification weights to match the age and sex distribution of the target population (18–44-year-olds living without HIV) in these communities using PopART Round 3 intervention service delivery data. In each round of delivering the PopART intervention, the aim was to list all households and the name, age and sex of all household members. As HIV status was not completely ascertained in this data, we used PC enrollment data to estimate HIV prevalence in these age-sex groups per community and applied these prevalences to estimate the age-sex distribution of 18–44-year-olds living without HIV for each community. Intervention data were only acquired from the intervention communities and so the estimated age-sex distribution was averaged across the intervention communities within each triplet and then used for each community in that triplet. The base design weights ${w}_{hij}$ were multiplied by the poststratification weights and trimmed such that final weights were within 4*IQR of the median of the weights for each community.

Weighted Poisson regression was used to estimate community-level HIV incidence for 18–44-year-olds, one for each community. The PopART intervention effect, measured during the period from the second year (PC12) until the end of the trial at the fourth year (PC36), was estimated using log-transformed values of community-level incidence estimates in an ANCOVA model including community-level baseline HIV prevalence, triplet indicator and intervention arm indicator, mimicking the primary analysis of the trial to obtain rate ratio comparisons of HIV incidence between arms A and C and arms B and C. This cluster-level approach is robust in analyzing cluster-randomized trials, providing valid results under a variety of circumstances and is preferred with a small number of clusters [3]. We note that weighting may increase within-community variance which in turn would increase the variance of the cluster means for the cluster-level analysis.

For comparison, we constructed unweighted HIV incidence measures in the PC0 analysis cohort enrolled in each community and used the same two-stage analysis method [2] of the PopART primary results, which adjusts for age and sex within the enrolled cohort but applied only to the PC0-enrolled cohort.

No imputation methodology was used in this analysis to adjust for item nonresponse: those enrolled participants that did not have at least two visits between the second and third year of follow-up were excluded. Post-stratification used addresses differences in the age-sex distributions from the PC and general community rather than more nuanced nonresponse patterns.

## Results

The analysis used follow-up from 16,286 PC0 participants enrolled at baseline and living without HIV at PC12 with at least one follow-up visit. Table 1 shows the community-level HIV incidence, weighted to represent incidence according to the age-sex distribution of HIV-uninfected 18–44 years-olds in each community. Incidence ranged from 0.42 to 2.51 events per 100 person-years, with a geometric mean across the communities of 1.16 events per 100 person-years. In nearly all communities, community-level incidence was at or below that measured among the individuals in the PC0 cohort, as expected, since men have increased representation in the community-level estimates and generally have lower incidence in this age range. We also see wider confidence intervals (CIs) for the community-level incidence estimates due to the use of weighting.

**Table 1:** PC0 cohort and Community-level HIV incidence (analysis period PC12-PC36)

Triplet	Arm	HIV incidence measured in PC0 analysis cohort, unadjusted (Estimate, 95% CI)	Community-level HIV incidence, estimated with age-sex standardization (Estimate, 95% CI)
Triplet 1	Arm A	2.13 (1.39, 3.27)	1.58 (0.98, 2.54)
Triplet 1	Arm B	1.00 (0.54, 1.85)	1.06 (0.52, 2.15)
Triplet 1	Arm C	1.28 (0.84, 1.97)	0.91 (0.51, 1.63)
Triplet 2	Arm A	1.75 (1.21, 2.54)	1.76 (1.06, 2.91)
Triplet 2	Arm B	1.11 (0.74, 1.65)	0.96 (0.54, 1.72)
Triplet 2	Arm C	1.33 (0.85, 2.09)	1.02 (0.61, 1.72)
Triplet 3	Arm A	1.41 (0.84, 2.39)	1.43 (0.77, 2.65)
Triplet 3	Arm B	1.63 (0.98, 2.70)	1.06 (0.59, 1.88)
Triplet 3	Arm C	1.57 (0.99, 2.50)	1.33 (0.77, 2.30)
Triplet 4	Arm A	1.72 (1.13, 2.61)	1.28 (0.81, 2.05)
Triplet 4	Arm B	1.20 (0.68, 2.11)	1.13 (0.54, 2.36)
Triplet 4	Arm C	2.08 (1.31, 3.30)	1.66 (0.99, 2.80)
Triplet 5	Arm A	2.33 (1.62, 3.35)	2.51 (1.52, 4.17)
Triplet 5	Arm B	1.84 (1.28, 2.65)	1.26 (0.77, 2.04)
Triplet 5	Arm C	2.25 (1.50, 3.39)	1.54 (0.97, 2.45)
Triplet 6	Arm A	1.40 (0.91, 2.15)	1.09 (0.64, 1.88)
Triplet 6	Arm B	1.18 (0.76, 1.83)	0.95 (0.55, 1.62)
Triplet 6	Arm C	2.30 (1.55, 3.40)	1.83 (1.04, 3.20)
Triplet 7	Arm A	0.60 (0.33, 1.09)	0.56 (0.26, 1.19)
Triplet 7	Arm B	0.43 (0.23, 0.79)	0.42 (0.18, 0.98)
Triplet 7	Arm C	0.68 (0.39, 1.17)	0.78 (0.38, 1.63)

**Fig. 1:**
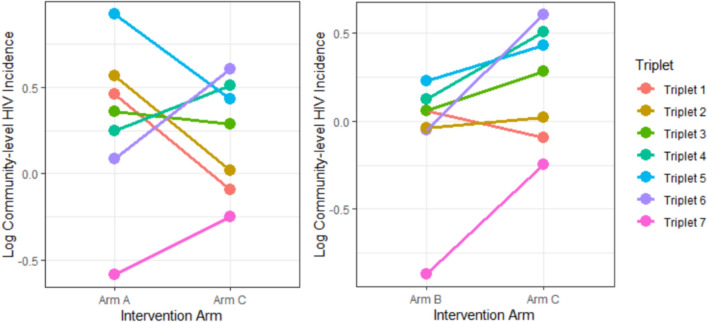
Log Community-level HIV incidence (analysis period PC12-PC36) compared for each intervention arm (A and B) to standard of care arm (C)

Figure 1 shows the intervention (Arm A and Arm B) log-community-level incidence estimates compared to standard of care (Arm C). In all but one of the matched triplets, Arm B communities have lower estimated incidence compared to standard of care; comparing Arm A to standard of care there is not a consistent intervention effect.

Table 2 displays rate ratios, confidence intervals and p-values for the PC0-enrolled analysis cohort, both unweighted and community-level analyses. The analysis based on community-level incidence shows a 25% reduction in incidence for Arm B communities compared to standard of care (Table 2; RR: 0.75, 95% CI: 0.56–1.02, p = 0.06) while Arm A communities show no significant difference in HIV incidence compared to standard of care (Table 2; RR: 1.08, 95% CI: 0.81–1.46, p = 0.56). By comparison, Table 2 also displays the unweighted analysis which shows a 28% reduction for Arm B compared to standard of care and no significant difference in Arm A compared to standard of care.

**Table 2:** Intervention effect based on unweighted (age-sex adjusted within cohort) and community-level HIV incidence (PC0-enrolled cohort, analysis period PC12-PC36)

	Unweighted PC0-enrolled Analysis Cohort		Community-level PC0-enrolled Analysis Cohort	
Comparison	Rate Ratio (95% CI)	p-value	Rate Ratio (95% CI)	p-value
Arm A vs. Arm C	0.96 (0.74, 1.25)	0.74	1.08 (0.81, 1.46)	0.56
Arm B vs. Arm C	0.72 (0.55, 0.93)	0.02	0.75 (0.56, 1.02)	0.06

## Discussion

The primary analysis of PopART reported a 30% reduction in Arm B community incidence compared to standard of care (p-value 0.006) and a 7% reduction in Arm A communities compared to standard of care (p-value 0.51)^5^. The PopART primary analysis estimated the intervention effect on people enrolled throughout the trial and had better power and precision compared to this re-analysis using weighting to estimate community-level HIV incidence.

The re-analysis presented here, using those enrolled at bassline, estimates a comparable intervention effect in the entire community. The differences seen compared to the previously published primary analysis, specifically comparing Arm A to standard of care, fall within the range of statistical uncertainty due to the small number of communities and the application of weighting to the analysis. While universal test-and-treatment has been implemented in Zambia and South Africa since 2016, this re-analysis helps strengthen the results from the PopART trial by accounting for the age-sex bias in the enrolled participant cohort.

Our finding that the community-level estimates of HIV incidence were lower than measured in the PC0 cohort, in which 72% of participants were women, was expected. Given that the intervention effect was strongest in men, who were underrepresented in the PC0 cohort, there was potential for the community-level analysis to show a stronger intervention effect. However, the intervention effects observed in both young men and young women in the cohort were small, and the estimates of intervention effects from the age-sex-adjusted community-level analysis were similar to those reported from the analysis of directly observed incidence in the cohort.

This re-analysis has limitations. We only used participants enrolled at baseline (74% of PC participants). The study continued to enroll participants during the second and third year of the HPTN 071 (PopART) trial who were included in the primary analysis of the effect of the intervention; however, design weights for these individuals were unknown. For post-stratification we used PopART Round 3 intervention data which were only available in intervention communities (Arms A and B communities). We assumed that the age-sex distribution from the intervention data were (1) stable from baseline through the analysis period and (2) representative of the matched standard of care communities. No other differences in population composition were directly evaluated. Lastly, we performed our analysis on those enrolled in the PopART trial who were not lost to follow-up, however there was no evidence this selection bias differed between intervention arms.

## Supplementary Information

**Additional file 1.**

## Data Availability

Data used in this study are available upon request, with no end date. This includes de-identified participant data with a data dictionary. Requests can be sent to HPTN-Data-Access@scharp.org. The study protocol is available here: https://pubmed.ncbi.nlm.nih.gov/24524229/

## Abbreviations

PC: Population cohort
HH: Household
IQR: Inter-quartile range
ANCOVA: Analysis of covariance
RR: Rate ratio
CI: Confidence interval
