# Supplementary material for: A survey weighted analysis of HPTN 071 (PopART) primary outcome of HIV incidence
Source: AIDS Res Ther. 2025 Mar 7;22:30. doi: 10.1186/s12981-025-00720-0 (PMC11887120; doi:10.1186/s12981-025-00720-0)

SUPPORTING INFORMATION

ACTIVE, RESERVE AND NOT SAMPLED HOUSEHOLDS

All but one community had at least 3125 households identified via the 2013 household census to fill the requirements of the active sampling list containing 3125 households. One community (#21) only had 2904 potential households and a decision was made to sample 1875 as the active list and 617 as the reserve list.

CONSTRUCTION OF POST-STRATIFICATION WEIGHTS

To obtain the final weights $w_{hij\_PS}$ used in this re-analysis, the base design weights $w_{hij}$ are multiplied by poststratification weights.

The post-stratification weights are constructed as the ratio of $p_{kt}$ and $\hat{p}_{kt}$. Using both the Round 3 Intervention Data and the baseline HIV status among the enrolled participants, $p_{kt}$ is the proportion of the people living without HIV in the *k^th^* age-sex strata in triplet *t*. We estimate $\hat{p}_{kt}$ as the sum of the base design weights for the PC0-enrolled people living without HIV in the *k^th^* age-sex strata in triplet *t* divided by the sum of the weights of those in triplet *t*. Thus,

$$\text{w}_{\text{hij\_PS}}\text{ }\text{=}\text{ w}_{\text{hij}}\text{ ×}\text{ }\frac{\text{p}_{\text{kt}}}{{\hat{\text{p}}}_{\text{kt}}}$$

Supplemental Figure 1: Age-sex proportions among PC0-enrolled cohort living without HIV, by community.


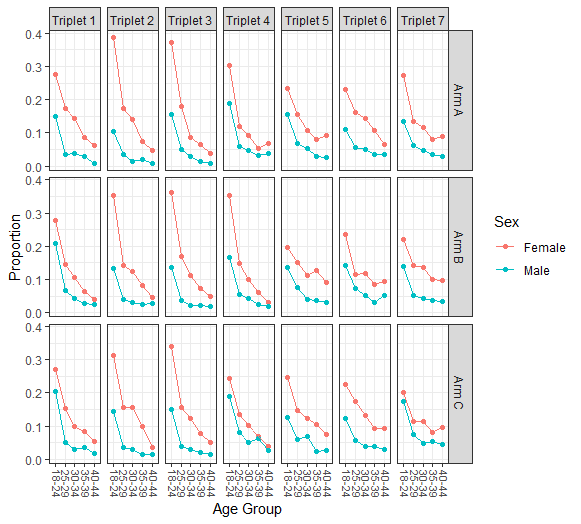


Supplemental Figure 2: Weighted Age-sex proportions among PC0-enrolled cohort living without HIV, by community.


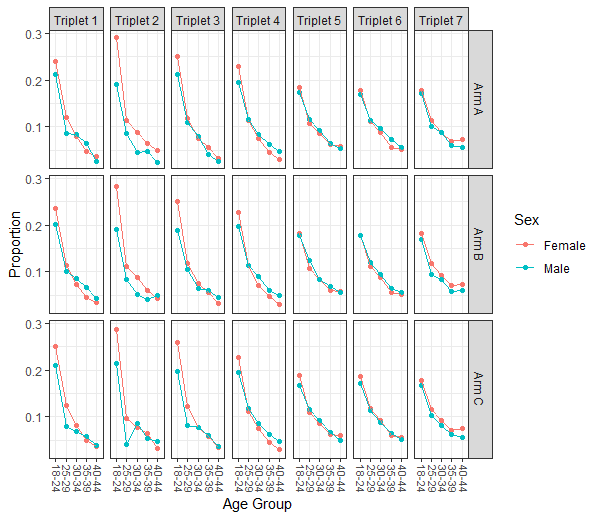


Supplemental Figure 3: Average weights for age-sex groups among PC0-enrolled cohort living without HIV, by community.


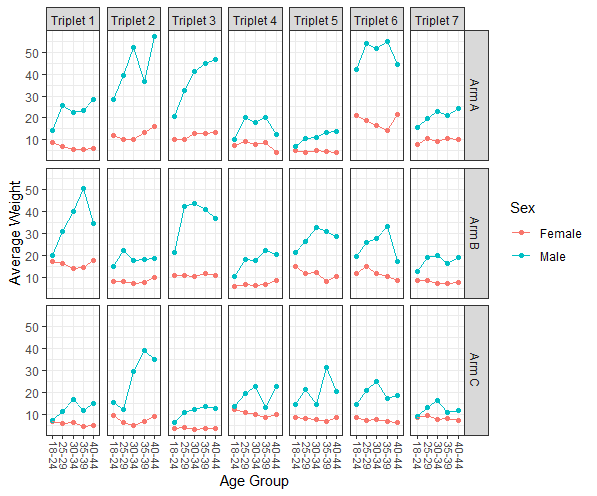

Supplement: Supplementary file 1 — Additional file 1. [file 12981_2025_720_MOESM1_ESM.docx]
